# Building a Decentralized Biobanking App for Research Transparency and Patient Engagement: Participatory Design Study

**DOI:** 10.2196/59485

**Published:** 2025-03-05

**Authors:** Ananya Dewan, M Eifler, Amelia Hood, William Sanchez, Marielle Gross

**Affiliations:** 1 Johns Hopkins University School of Medicine Baltimore, MD United States; 2 de-bi, co. Baltimore, MD United States; 3 Johns Hopkins Berman Institute of Bioethics Baltimore, MD United States

**Keywords:** mobile health, mHealth, application, smartphone, digital health, digital intervention, participatory design, biobanking, research transparency, donation, patient-derived biospecimens, plain language communications, patient education

## Abstract

**Background:**

Patient-derived biospecimens are invaluable tools in biomedical research. Currently, there are no mechanisms for patients to follow along and learn about the uses of their donated samples. Incorporating patients as stakeholders and meaningfully engaging them in biomedical research first requires transparency of research activities.

**Objective:**

In this paper, we describe the use of participatory design methods to build a decentralized biobanking “de-bi” mobile app where patients could learn about biobanking, track their specimens, and engage with ongoing research via patient-friendly interfaces overlaying institutional biobank databases, initially developed for a breast cancer use case.

**Methods:**

This research occurred in 2 phases. In phase 1, we designed app screens from which patients could learn about ongoing research involving their samples. We embedded these screens in a survey (n=94) to gauge patients’ interests regarding types of feedback and engagement opportunities; survey responses were probed during 6 comprehensive follow-up interviews. We then held an immersive participatory design workshop where participants (approximately 50) provided general feedback about our approach, with an embedded codesign workshop where a subset (n=15) provided targeted feedback on screen designs. For phase 2, we refined user interfaces and developed a functional app prototype in consultation with institutional stakeholders to ensure regulatory compliance, workflow compatibility, and composability with local data architectures. We presented the app at a second workshop, where participants (n=25, across 9 groups) shared thoughts on the app’s usability and design. In this phase, we conducted cognitive walkthroughs (n=13) to gain in-depth feedback on in-app task navigation.

**Results:**

Most of the survey participants (61/81, 75%) were interested in learning the outcomes of research on their specimens, and 49% (41/83) were interested in connecting with others with the same diagnosis. Participants (47/60, 78%) expressed strong interest in receiving patient-friendly summaries of scientific information from scientists using their biospecimens. The first design workshop identified confusion in terminology and data presentation (eg, 9/15, 60% of co-designers were unclear on the biospecimens “in use”), though many appreciated the ability to view their personal biospecimens (7/15, 47%), and most were excited about connecting with others (12/15, 80%). In the second workshop, all groups found the app’s information valuable. Moreover, 44% (5/9) noted they did not like the onboarding process, which was echoed in cognitive walkthroughs. Walkthroughs further confirmed interest in biospecimen tracking, and 23% (3/13) had confusion about not finding any of their biospecimens in the app. These findings guided refinements in onboarding, design, and user experience.

**Conclusions:**

Designing a patient-facing app that displays information about biobanked specimens can facilitate greater transparency and engagement in biomedical research. Co-designing the app with patient stakeholders confirmed interest in learning about biospecimens and related research, improved presentation of data, and ensured usability of the app in preparation for a pilot study.

## Introduction

### Background

Samples of blood, tissue, urine, and other biological products, collected from patients during a medical procedure, are invaluable tools in biomedical research. Reviews estimate that >600 research biobanks exist in the United States, housing tens of millions of samples from tens of millions of patients [1,2]. Biospecimens are used in a vast array of foundational, translational, and clinical research [3-6]. Research on biospecimens shows exceptional promise in studying cancer and developing new cancer treatments [4,7,8]. Cutting-edge specimen preservation methods offer high-fidelity disease models for genomic analysis, high-throughput drug testing, advanced imaging, and more.

**Figure 1:**
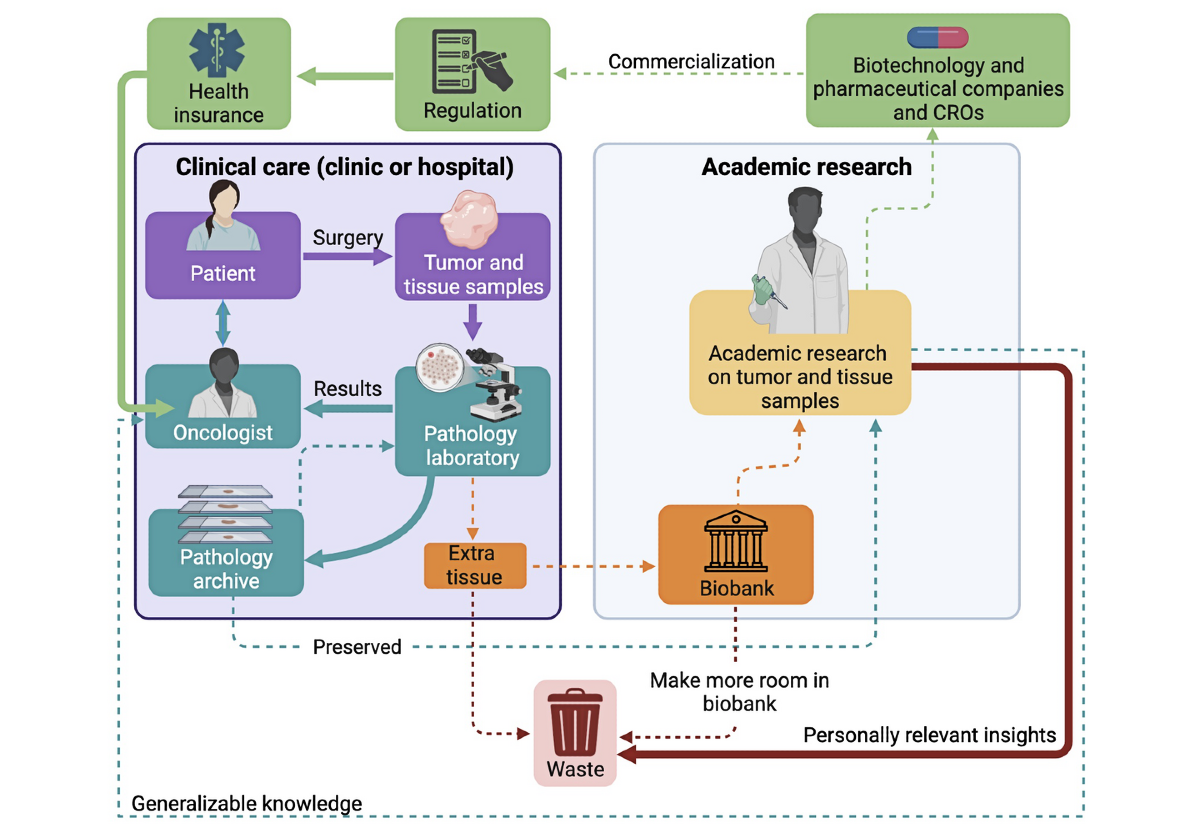
Biobanking ecosystem: biospecimen supply chain from clinical by-products to institutionalized assets. In the current biobanking paradigm, personally relevant insights and extra tissue are often wasted, representing lost opportunity to advance patient care and maximize value from donated biospecimens. CRO: contract research organization.

Typically, leftover surgical samples are collected after a 1-time informed consent process per the federal Common Rule in the United States*,* where patients receive information about the proposed research [9]. The terms of this consent include no expectation to follow up with pertinent research results after the form is signed and samples are collected [10]. In the transition from clinical by-product to banked research sample, patient identifiers are removed from biospecimen data [11]. Deidentification is meant to protect patient privacy by removing identifiable information; however, its conditions preclude scientists from sharing follow-up information about if, when, and how they use biospecimens for research [12]. Scientists receive deidentified biospecimens from the biobank and have no mechanism to directly communicate personally relevant insights or engage with the people whose samples they have received (Figure 1) [10]. Thus, while clinical results from pathology reports are communicated to patients, information on how their biospecimens are used and associated research findings are never shared.

Incorporating patients as stakeholders and meaningfully engaging them in biomedical research is increasingly recognized as critical to more ethical and effective scientific advancement [13]. Patients’ lived experiences can help guide research questions, priorities, and methodologies, ensuring that the resulting knowledge aligns with their and their community’s priorities. Various innovative technologies and techniques have enhanced patient engagement in research [14,15]; for instance, the James Lind Alliance method facilitates collaboration among clinicians, the public, and additional stakeholders, enabling their opinions to converge and inform the establishment of research priorities [16,17]. Digital platforms that assemble citizens from different backgrounds to share experiences and actively codevelop innovative health strategies have been proposed [18]. However, improving transparency surrounding the nature and extent of research activities is a prerequisite to achieving true patient engagement.

Decentralizing biobank information and empowering biospecimen donors with knowledge about how their samples are used lays the foundation for fostering trust and transparency in biospecimen research [19]. This is particularly vital in rebuilding trust with communities considered marginalized, which have historically been subject to exploitation and unethical research practices [20]. Building trust lays the foundation for further participation by diverse communities and ensures that research endeavors are accountable to those whose biospecimens make the research possible [21].

In previous work, we have demonstrated that there is unmet patient demand for increased transparency surrounding biospecimen research use [22]. We surveyed patients with breast cancer (n=109) about their interest in learning about biobank research and their individual biospecimens [22]. This survey measured patient interest in a broad range of information that they could theoretically learn about research on their samples. Notably, survey respondents were interested in learning information that is readily available within in local biobank and research databases, such as whether their samples had been used in research (66%), details about research conducted on their samples (53%), and the ability to see images of their biospecimens if available (68%).

### Objectives

To address the unmet need for greater transparency and engagement between biobanks and patients, we sought to build an initial decentralized biobanking app—“de-bi”—to serve as a patient’s “window” into an established biobank database. We aimed to build a composable platform that would be accessible and valuable to patients while being compliant with existing regulations, composable with data architectures and compatible with established workflows [23]. By viewing biobank data, patients can be empowered to “track” what happens to their biospecimens [24]. A patient-facing mobile app could overlay and display existing data, serving as a technological tool for increasing the transparency of research activities and as a mechanism for directly engaging with patients who have contributed to research, all within the current frameworks of consent and deidentification. Composability with institutional databases would also serve as a source of efficiency, easing the burden of the initial manual data transfer to an app interface and setting up easier future automation and scalability. This tool could then be piloted to measure its impact on patient participation, understanding, and attitudes about biobanking and biospecimen research and begin to answer outstanding empirical questions about effectively fulfilling obligations of transparency and engagement.

Including patients in the design phase was imperative to the future pilot’s success. Before the tool was deployed, we sought to ensure that it was interesting, accessible, understandable, valuable, and easy and ideally pleasant to use. Here, we describe the process and results of using participatory design methods to define, design, and deliver a mobile app that enables patients to learn about their biospecimens collected for research, the status of the biospecimens’ use in research protocols, and overall biobank collections, as demonstrated in a breast cancer biobank setting.

## Methods

### Overview

This multiphase participatory design study was conducted among patients of a breast cancer clinic at a large US academic institution. It consisted of surveys of patients (n=94), six semistructured in-depth follow-up interviews with five survey respondents (5/94, 5%), immersive design workshops with patients (workshop 1: approximately 50 participants, focusing on a subset of 15 codesign participants), workshop 2: n=25), and cognitive walkthroughs with individual patients (n=13) [25]. The data being presented represent a subset of broader participant engagement, including additional workshops and encounters with the local community of patients with breast cancer.

**Figure 2:**
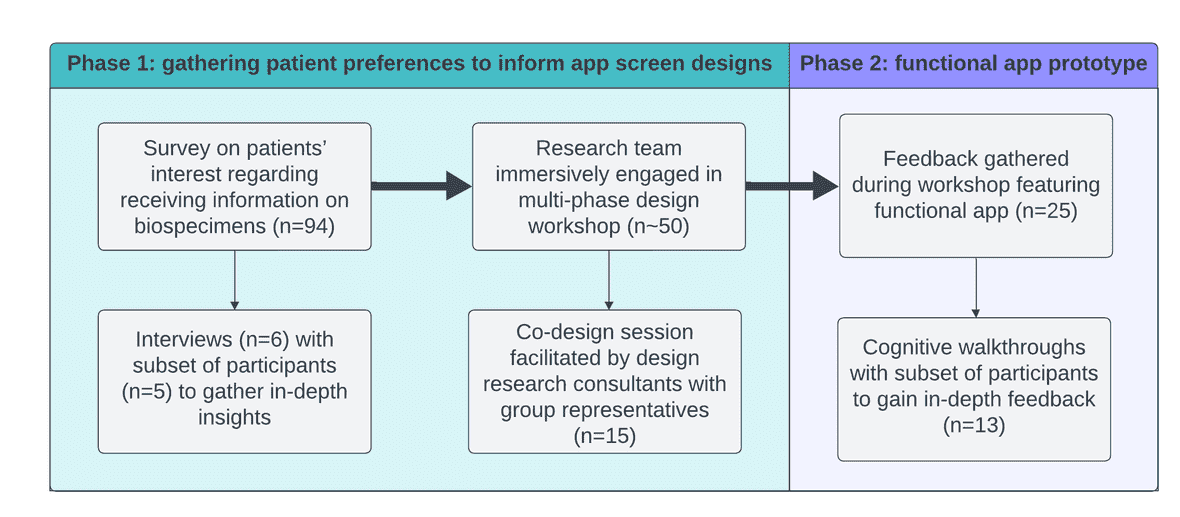
Overview of study methods.

This research occurred in 2 phases (Figure 2). In phase 1, we designed smartphone screens containing different information that patients could learn about biobank and research activities involving their samples. Embedding these screen designs in a survey, we sought to gauge patients’ interest in receiving information about research or their biospecimens. We engaged a subset of survey respondents in short web-based interviews to discern their views on the importance of having this information and their opinions on its presentation and design. We held a design workshop at which participants gave feedback on the screens and suggested improvements.

For phase 2, we refined the user interfaces and developed a functional app prototype. As we developed the app, we consulted institutional stakeholders to enhance compatibility with regulations and composability of core app features, with one another and with local data architectures. We then distributed the app at a second design workshop, where participants shared thoughts on the usability and design of the app. In this phase, we conducted cognitive walkthroughs with individual participants to observe their success in using the app and to gain in-depth feedback on its functionality [26].

### Phase 1

#### App Screen Design

We used Whimsical and Figma for the initial wireframes and Flutter and Adalo for prototyping to develop the initial screen designs [27,28]. The content of these designs was informed by previous engagement with this patient population and limited by the content available within institutional biobank or research databases and publicly accessible research content. Previous survey results identified important data points to include in our patient-facing display and informed its design [22]. Working with investigators on the biobanking research protocol, their data managers, and the University of Pittsburgh Institutional Review Board, we selected data points that would be feasible and acceptable to return to patients in a future, functional solution. We then created a sample screenshot presenting a model set of samples and the corresponding hypothetical data points.

**Figure 3:**
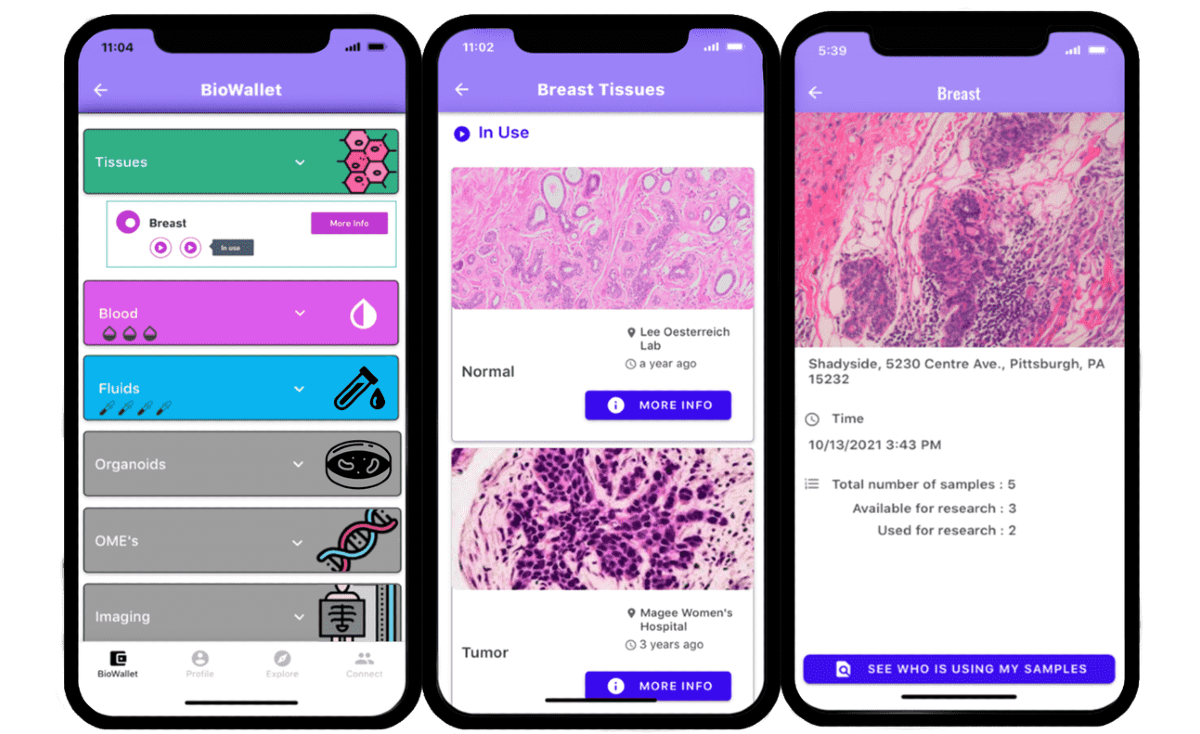
The 3 “biowallet” screens, as developed in Adalo and featured in the survey, presenting different categories of specimens using accordion unfolding and individual sample–level details.

We designed 3 initial app screens presenting information about a hypothetical user’s collection of biospecimens in the biobank (Figure 3). These screens were meant to be viewed sequentially, with increasing specificity of information presented about individual biospecimens in the biobank. The interface was inspired by the term “biobanking” and designed to resemble many banking apps where users first view a summary of their accounts and can click through to increase specificity about each one. Our first screen contained a summary list of a hypothetical individual’s inventory of donated samples, including pictures of each. The second screen displayed high-level demographic information about each individual biospecimen: an image, its location, and the date it was collected. The third and final screen showed more details about each individual sample, such as its ID number, the medical procedure from which it was collected, the number of the freezer where it was stored, and more.

**Figure 4:**
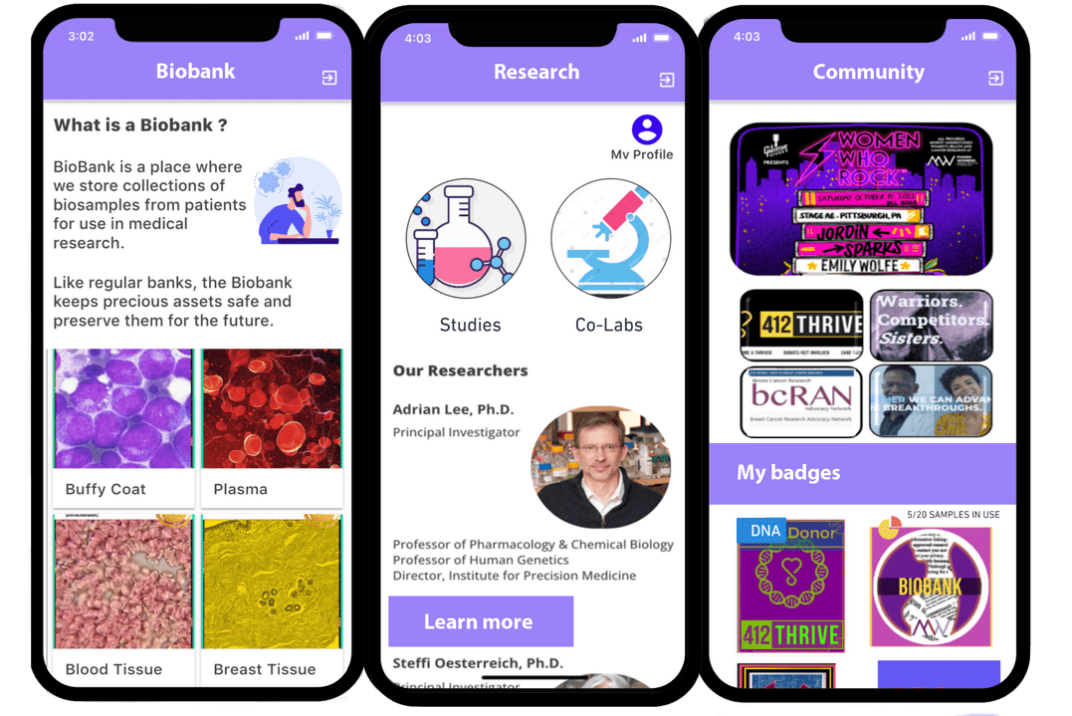
Biobank, research, and community screens. These screens, developed with Adalo, illustrate features explored in surveys, interviews, and workshops, including opportunities to learn about biobanking and specific specimens, follow scientists and learn about individual research programs, and connect with fellow donors and scientists.

A fourth screen was created for potential users to learn about the biobank collection as a whole (Figure 4). This screen, called “connect,” showed a list of subcommunities of biobank donors sorted into groups by their diagnosis code, including summary statistics indicating how many donors to the biobank shared a diagnosis (eg, invasive ductal carcinoma). The design suggests that users might be members of ≥1 of these groups (called “labs”) and might use their membership to “connect” with other users, patients, or scientists.

#### Surveys and Semistructured Interviews

After developing the initial app screens, we embedded them into a survey to gather further evaluation and feedback from patients [29]. We conducted the survey in 2023 with a convenience sample, recruiting patients via flyers posted in breast care clinics across the health system. Participants took the survey on their own devices. IP addresses were used to identify duplicate entries from the same users. At the end of the survey, participants were invited to participate in an optional follow-up semistructured interview to discuss their survey answers. Survey content was iterated and rotated as app screens were finalized and in response to saturation of data from responses and follow-up interviews. Thus, survey data are presented with the denominator reflecting the number of participants who received each specific question and chose to reply. In total, 94 participants completed the survey, of whom 5 (5%) participated in the follow-up interviews (one participant was interviewed twice; once before and once after her cancer diagnosis). Demographics of the survey respondents, including age, race, and cancer stage, are presented in Table 1. The “Unknown” category represents participants who chose not to answer that question.

The survey asked participants their level of interest in the information presented on the app screens. After viewing the “biowallet” screens, participants were asked which of the presented data points were of interest to them. After viewing the “connect” screen, patients were asked whether identifying and belonging to a subcommunity of donors to the biobank would be of interest to them and, if so, how they might want to use the app to connect with others; for example, they were asked whether they were interested in connecting with researchers who used their samples from the biobank and, if so, what information they would like to exchange with them. They were also asked about their interest in connecting with other, similar patients who might be members of the same subcommunity (eg, have the same diagnosis code) and, if so, how.

In the follow-up semistructured interviews, participants and members of the research team viewed the participants’ survey responses via screen sharing on an audio-recorded web-based call. The participant’s survey responses were walked through one at a time, and the participant was asked to go into further detail about their reasoning for their answers. These interviews were meant to validate the survey questions by identifying any confusing language or images and to gain a deeper understanding of the motivations, preferences, interests, and concerns of participants. Each interview lasted 35 to 45 minutes. Interview participants were selected to represent key subsegments of the survey population, for example, age, education, and time from diagnosis.

Survey responses were analyzed, and summary statistics are presented. Transcripts of the follow-up interviews were coded for emergent themes in response to each set of screens. The results from both analyses informed changes to the presentation of biobank data and adjustments to the design of the screens to meet participant preferences and clarify content.

**Table 1:** Socioeconomic and clinical characteristics of survey respondents (n=94).

Demographics				Participants, n (%)^a^
**Socioeconomic characteristics**				
	**Age (y)**			
		<40	12 (13)	
		40-49	32 (34)	
		50-59	22 (23)	
		60-69	14 (15)	
		≥70	11 (12)	
		Unknown	3 (3)	
	**Race**			
		White	64 (68)	
		Other (including multiracial)	10 (11)	
		Unknown	20 (21)	
	**Household income (US $)**			
		<35,000	12 (13)	
		35,000-69,999	16 (17)	
		70,000-99,999	17 (18)	
		≥100,000	27 (29)	
		Prefer not to say	2 (2)	
		Unknown	20 (21)	
	**Highest education**			
		Did not graduate high school	1 (1)	
		High school	6 (6)	
		Some college	9 (10)	
		Associate degree	10 (11)	
		Bachelor’s degree	21 (22)	
		Master’s degree	17 (18)	
		Doctoral or professional degree	9 (10)	
		Unknown	21 (22)	
**Clinical characteristics**				
	**Breast cancer diagnosis**			
		Yes	56 (60)	
		No	35 (37)	
		Unknown	3 (3)	
	**Cancer stage**			
		Unsure	5 (5)	
		0 or precancer	4 (4)	
		I	22 (23)	
		II or III	23 (24)	
		IV	2 (2)	
		Unknown	38 (40)	
	**Time from diagnosis**			
		≥5 y ago	11 (12)	
		>1 and <5 y ago	31 (33)	
		≤1 y ago	14 (15)	
		Unknown	38 (40)	

^a^Due to rounding, not all percentages sum to 100%.

#### Design Workshop 1

We sought feedback on the “biowallet” screens, the “connect” screen, and 2 additional screens: “explore” and “profile.” The “connect” feature was expanded to include 3 screens showing a list of all “lab” groups and subcommunities, a description and membership details of 1 specific “lab,” and an action button to “add my samples” to a “lab.” The screen called “explore” contained additional biobank-level data about the collection of biospecimens. The last addition, “profile,” contained a hypothetical user-chosen avatar to represent themselves, as well as a collection of hypothetical achievement badges representing activities that users might be able to complete both in the app (“claimed my biowallet”) and in research participation (“signed a consent form”). It also contained a progress bar indicating the users’ completion of in-app tasks and achievements. We presented the second iteration of the screens to approximately 50 members of a local support group for patients with breast cancer in an embedded participatory design workshop. Members of the research team presented the screens, gave a short overview of their contents, and explained the overall intent of decentralized biobanking as well as the purpose of the dedicated app codesign workshop.

Participants provided feedback about the overall approach during a three-day participatory engagement event where the research team presented to and engaged with the local breast cancer patient and previvor population in an immersive setting. In this paper, we focus on describing results for 15 group representatives who attended a professionally facilitated in-person codesign workshop where they provided detailed feedback on individual app screen designs. Participants were given printed images of the screens, along with stickers—thumbs up or a heart to signify something they liked, a question mark to signify something they were confused by, and an exclamation point or a frowning face to signify something they did not like. Participants were asked to discuss the screens as a group and to place stickers on the printed images, either to signal their reaction to the entire screen or specific parts of the image. Some groups wrote on the images or in the margins to elaborate on their responses. As not all participants responded to all app screens, workshop data for all subsequently described workshops and cognitive walkthroughs are presented with the denominator reflecting the number of participants who provided written feedback on a given screen, page or design element.

The printouts were collected after the workshop, and the research team performed a content analysis of the sticker placements and notes left by participants. The identified themes were considered along with design research synthesis from expert consultants, as well as analysis of survey responses, semistructured interviews, and immersive engagement. Taken together, these themes informed further design changes that were then incorporated into a functioning mobile app prototype.

### Phase 2

#### App Prototype Development

We developed a functional mobile app using Flutter [30]. The front-end patient-facing interfaces contained 4 sections: “biowallet,” “explore,” “connect,” and “profile.” We also developed a backend data architecture in which real biobank data could be imported into the app so that users could see personalized, real-time information about their biospecimens contained in the biobank collection. During the app’s development, we consulted with institutional stakeholders to ensure regulatory compliance and compatibility with existing data resources to minimize burdens on researchers and data managers. Elsewhere, we describe a “digital honest broker” approach that enabled pilot participants to be connected directly to their own deidentified biospecimens [31].

#### Design Workshop 2

We held a second design workshop with a second local advocacy and support group for patients with any cancer type. A total of 25 participants attended. Members of the research team presented the app screens, gave an overview of their contents, and explained the intent of the workshop. Participants were then instructed to access the app on their personal devices and given a sample log-in leading to a deidentified demonstration account with biobank-level data and individual biospecimen data populated. Participants were given a sheet of paper with three columns—“Things I Liked,” “Things I Didn’t Like,” and “Didn’t Meet Expectations”—and were asked to write corresponding reactions as they explored the app freely by themselves or with one or two other participants. The papers were collected from nine small groups. Content analysis was performed to identify common areas of interest, enjoyment, displeasure, and dissatisfaction.

#### Cognitive Walkthroughs

During the second design workshop, members of the research team invited individuals at random to participate in a cognitive walkthrough to evaluate the usability of the application. Of the 25 workshop participants, 6 (24%) completed the walkthrough exercise. Participants were asked to perform several in-app tasks and seek specific information presented in the app while narrating out loud what they were doing. Members of the research team observed them using the app and took notes about their narration, evaluating their success in completing the assigned tasks.

We held seven additional cognitive walkthroughs with users recruited from participants in the phase 1 survey who agreed to be contacted about future research. These participants were patients at the collaborating health system and were guided through the onboarding process that connected them to their own biospecimen information, if any existed. All users were able to see the “explore” and “profile” screens. Users who had also consented to the biobanking protocol (which was manually verified by the research team) were able to see the “connect” screen. If the user had samples in the biobank, they could see the “biowallet” screen populated with real data about their samples. Participants were led through the same cognitive walkthrough and asked to complete tasks and seek out specific information while they narrated their process as researchers observed and took notes.

Cognitive walkthroughs measure usability by collecting qualitative, experiential data from users directly. Researchers can infer usability by noting whether the users understand the task and are trying to achieve the right outcome, whether they take the appropriate action to achieve their intended outcome, and whether they correctly identify their success or the information sought from them. By having a mix of participants viewing uniform, hypothetical data and those viewing personalized data, we were able to gain generalized insights into usability, as well as insights into the personal value and added joy or frustration when engaging with one’s own data. Both sets of participants were asked to create an account; perform the necessary authentications to populate their biospecimen data; identify how many of their samples had been used in research and how many were available; and to find a protocol that had used their samples, or, if their samples were unused, to find a protocol that studied their particular diagnosis.

### Ethical Considerations

This study was approved by the University of Pittsburgh Institutional Review Board and quality improvement committees (IRB22010118, IRB22020035, and QRC3958). Participants provided informed consent before engaging with the study. The voluntary nature of the study was emphasized, including that their decision to participate in the study would have no impact on their care. Participants were able to skip questions or withdraw at any time. To safeguard privacy, data were stored on a secure institutional server, with survey responses deidentified and stored separately from personal information. Survey participants were paid US $10 for their participation upon completion. A subset of survey participants who agreed to participate in the follow-up interview were paid an additional US $20.

## Results

### Phase 1

#### Surveys

In reaction to the “biowallet” screen, participants were asked about their interest in using such an app to track their biospecimens donated to research. Of the 89 respondents, 20 (22%) were extremely interested, 22 (25%) were moderately interested, 24 (27%) were neutral or not sure, and 23 (26%) were not interested. Survey questions presented potential app features related to sample tracking, research feedback, and community engagement (Table 2), and follow up questions drilled down on preferences regarding specific design elements and user priorities.

We next assessed participants’ interest in receiving updates regarding how, and by which researchers, their samples were used (Table 2). Participants (61/81, 75%) were most interested in learning “the outcomes of studies that use my tissues, like publications or new discoveries.” Of these 61 participants, 14 (23%) provided qualitative details in optional open-ended follow-up questions, describing how or why they were interested in learning about research on their samples. For example, one respondent noted “I believe research participants should have full access to the data that is collected from them...The idea that my genetic data is setting in a lab for a variety of research studies, but I have no access to any of that data or the related information generated by that data is not fair or in my best interest.”

The second highest area of interest concerned learning the status of their biospecimens regarding whether they had been used in research (45/81, 56%). Only 23% (19/81) of the participants were interested in seeing how many samples they had compared to other patients with the same diagnosis. Other data points of interest were the medical procedure from which their sample was collected (12/27, 44%), the current location of their sample (11/27, 41%), and collection details (when, volume, etc; 10/27, 37%).

Subsequently, we asked participants about their broader preferences for updates from researchers using their biospecimens with respect to scientific findings, new developments, and modes of communication (Table 2). Participants (47/60, 78%) expressed the greatest interest in receiving patient-friendly summaries of scientific information. Updates on new drugs or tests that become commercially available (39/60, 65%) and findings that may inform care (38/60, 63%) were also highly valued. Fewer patients were interested in updates on new publications (28/60, 47%), patient-friendly videos (22/60, 37%), study enrollment numbers (19/60, 32%), and new research grants (15/60, 25%).

In reaction to the “connect” screen, respondents expressed interest in connecting with other patients. Participants were most interested in connecting with patients with a similar type of breast cancer to theirs (41/83, 49%) and patients in their age group (36/83, 43%). Of the 83 respondents, 24 (29%) were not interested in connecting with any others. Table 2 details additional areas of interest.

**Table 2:** Interest in potential app features: biospecimen tracking, research updates, and community engagement^a^.

App feature interest			Survey responses, n (%)
**Sample tracking (“biowallet”) features (n=81)**			
	Outcomes of the studies that use my tissues, like publications or new discoveries	61 (75)	
	Learning if/when my biospecimens are shared with researchers	45 (56)	
	Seeing how my own samples look under the microscope	35 (43)	
	Learning about the individual researchers who are studying my biospecimens	27 (33)	
	Seeing how many samples I have compared to patients with the same diagnosis as me	19 (23)	
	None	12 (15)	
	Other	3 (4)	
**Research updates (“scientist labs”) (n=60)**			
	A patient-friendly summary of the scientific information	47 (78)	
	If there are new drugs or tests discovered that become commercially available products	39 (65)	
	If there are findings that might help my doctor and I make decisions about my care	38 (63)	
	When new publications come out (with access to that publication)	28 (47)	
	Short patient-friendly videos describing the research	24 (40)	
	How many patients are included	19 (32)	
	If there are new grants awarded to that research	15 (25)	
**Community engagement (“donor labs”) (n=83)**			
	Similar type of breast cancer	41 (49)	
	Patients in my age group	36 (43)	
	Patients in the same research study	30 (36)	
	Similar stage of breast cancer	25 (30)	
	None	24 (29)	
	Similar genetic risk of cancer	18 (22)	
	Patient advocates	12 (14)	

^a^These are not the only survey questions posed, but represent three key areas that were included in the survey. Where appropriate, questions were iterated to include screenshots of the app designs or concepts as they were refined.

#### Semistructured Interviews

While learning the outcomes of research on their biospecimens was identified as a high priority, concerns arose in the follow-up interviews. Of the 5 participants, 3 (60%) expressed concern about imposing additional burdens on busy scientists. They had expressed interest in learning about research on their specimens but clarified in interviews: “only if it doesn’t take away from the work” and “I don’t want to bother researchers, who are very busy” (Table 3). However, participants valued the opportunity to learn more about their samples; participant 3 expressed how specimen tracking can provide transparency about “what it’s [biospecimen] being used for,” implying how transparency may build trust.

In the follow-up interviews, 3 (60%) of the 5 participants expressed an interest in and need for communities that connect patients to research in general, stating that existing patient organizations were focused on emotional support, living with cancer, and survivorship rather than on biomedical research or the latest advances in treatments. Participant 2 expressed frustration with the lack of medical and scientific expertise in many patient support groups (Table 3): "...It’s frustrating sometimes to get the kind of information you’re looking for as a patient...with metastasis."

One participant undergoing diagnostic workup for cancer expressed interest in an additional support group, but without the granularity of connecting with other patients involved in the same studies. In contrast, one interview participant with a longer duration of cancer survivorship expressed that they did not have a need for additional patient connections. Multiple participants (3/5, 60%) expressed an interest in contributing additional information to scientists to provide additional context to their biospecimens. This included integrating data from genetic tests (eg, 23andMe), wearable devices (eg, Fitbit), and personal medical histories to support scientists. The motivation for doing so seemed to stem from the opportunity to “help...the scientific community, the research for cancer” (Participant 1) and elucidate “genetic links to breast cancer” (Participant 5). Participant 3 expressed interest in being informed about “a list of studies” they might qualify for, indicating a proactive approach to supporting biomedical research.

**Table 3:** Qualitative interview insights. The interview quotes are organized around feature categories of biobanking education, specimen tracking, connecting with others, and dynamic data sharing. Key standout phrases are italicized,

Proposed features (“naming convention”) and interview quotes			Emerging themes
**Learn biobanking (“biobank”)**			
	“I *totally geek out* on this stuff, so as much as you guys are willing to share and whatever level detail, I’m all about it.” (Participant 1, Interview 2) “I would want to know outcomes of the studies. I am kind of *a science geek*, like that’s my interest, it’s not the field that I’m in whatsoever, and I was not educated in that, but I’m very interested. I’ve read a lot about science and especially like bioscience, so I would say, I would also like to know outcomes, with the studies that use my tissues.” (Participant 3)	Valuing scientific knowledge and approaches	
	“Whatever you guys are willing to share is perfectly fine...*I just don’t expect that detailed level of personal information* about the researchers, but if they’re willing to share...it’s fascinating...it’s just not one of the absolutely necessary things, I feel.” (Participant 1, Interview 2) “*I don’t expect to have the ability,* or even the need to identify specific researchers who I can go to. It’s like as many people can use that tiny little tumor that you guys got out of me as possible it’s fine, so what, for whatever purpose and whoever needs it, for whatever reason, is fine.” (Participant 1, Interview 1) “It was my pre-op...my surgeon, and a nurse, she just had a whole pile of papers. It [the biobanking consent form] *was just one more paper she needed to get signed.*” (Participant 3)	Recognized unfamiliarity with biobanking, limited expectations and agency of donors within in current model	
**Specimen tracking (“biowallet”)**			
	“I don’t need to know about all the researchers and everyone who’s touching it. Just in general, like *what you’re looking at and...what you’re doing with it.*” (Participant 1, Interview 2) “I don’t feel the need to have control over any of that stuff—You guys can do with it what you see fit.” (Participant 1, Interview 1) “I *don’t want to bother researchers,* who are very busy.” (Participant 2) “It would be interesting to see my samples, and my tissue, but not necessary, I guess. So, sort of like an added bonus.” (Participant 5)	Balancing gaining personalized insights versus resource constraints for scientists	
	“I mean, worst case scenario, like these are my few... last few minutes on earth... It would bring meaning...I’m *suffering but I’m suffering for a reason.*” (Participant 4) “...People are fearful of having their information being used in nefarious ways; I think *this is a way of being super transparent and also involving the candidate*...like it’s personal, it’s something [very] personal, especially for women with breast tissue, very personal.” (Participant 4)	Recognition for personalized contributions as a source of purpose and meaning	
**Community engagement (“labs”)**			
	“Probably in the lines of a support group kind of thing, it would be kind of neat to see...what other people were experiencing and going through, but not necessarily with regards to the study itself.” (Participant 1, Interview 1) “You have questions in there, ‘Do you want to be connected with other donors?’ and [in the survey] I said no, ‘I don't use social media,’ but I would use this, and perhaps if I do have to get surgery or I...perhaps [have cancer]... *it’s like people who have dogs from the same litter, you know it’s like, ‘How’s your pup doing?’*” (Participant 3) “You know, now that I am talking this through, I would also...need...the type, the stage, the genetic risk of patients in my age group. I might want any one of those options. I am thinking futuristically now that it’s become an actual reality or possibility...*I would be more open to connecting to others...beyond the same research.*” (Participant 3)	Some felt existing support groups were adequate, while others sought camaraderie and citizen science	
	“One of the limitations with the patient advocacy groups that I’ve been in so far is that *there is limited expertise in the groups*...so it’s frustrating sometimes to get information you’re looking for as a patient...for example, drugs for preventing metastasis...what are the researchers recommending at this point [for people like me].” (Participant 2) “I think the research is important and I’m just interested in knowing what research is going on, just because I think it’s interesting, but *I wouldn’t necessarily need to know what’s happening with other patients* and their stuff.”(Participant 5) “I want to help, do whatever is possible to help research continue, but I don’t feel the need to use something like this to connect with other people [i.e., patients].” (Participant 5)	Unmet need for direct connection with scientists, as opposed to other patients	
**Data sharing (“profile”)**			
	“I have the ancestry/23andMe, I do Fitbit stuff, so yeah, that’d be very cool to be able to incorporate all that...Anything that would help in any way... the scientific community, the research for cancer, anything, advanced in any way, for whatever reason, whatever purpose.” (Participant 5) “There’s so much that’s not known in terms of...genetic links to breast cancer...*I would share more information if it would help.*” (Participant 5) “I would like to see a list of studies that I might qualify for, *I would provide details about my medical history or treatment.*” (Participant 3) “[If] you guys need additional information or stats or blood or anything from me, I’m more than happy to help.” (Participant 1, Interview 1)	Eager to contribute more specimens and data	
	“I keep throwing out those ads for...‘All of us’ *because they won’t give you anything...that’s my reason for not participating*...[since] you have all my genetic information, you have no idea where it’s going to be five years from now...But you won’t let me have any access to it.” (Participant 2) “I think it’s brilliant and I also think that *more people would participate if this were available*...Because I’ve had opportunities in the past, and...the biggest question that I have, and other people have, even when we’re asked, ‘Would you donate organs’...is the lack of knowledge about what it’s being used for...that’s what makes people suspicious and, especially, in an era where we both have like open source, you can find out information about everyone.” (Participant 3)	Demand for greater reciprocity and accountability for data exchange	

#### Design Workshop 1

Participants of the first design workshop (n=69) confirmed their interest in the concept of the app as well as the content of the app screens and provided valuable feedback on its presentation. A subset of 15 participants who participated in a dedicated codesign session documented their reactions to individual app screens. The “biobank” screens were appreciated by participants who liked the ability to view the entire collection of one’s personal samples (7/15, 47%), as well as the sample-level details under the individual “biowallet” (4/15, 27%), with several emphasizing the linkage between samples and other medical or genetic data. Some were confused or upset by the technical details presented about each individual sample and about some of the terminology related to their biospecimens (eg, questioning what it means for a sample to be depicted as “in use”; 9/15, 60%). Of 15 co-designers, 4 (27%) noted that they would have preferred their samples to be organized chronologically, corresponding to their lived experience of appointments and procedures as specimen collection events, rather than initially stratified by sample type (tissue, blood, urine, organoids, etc.). For example, one co-designer emphasized: “Everything is timeline for us [patients with cancer].”

Moreover, 80% (12/15) of co-designers provided positive reactions to the “connect” screens, where they hearted the framing of virtual “lab” groups as ‘patient-led’ (5/12, 42%) and appreciated that there seemed to be actions that a user could take, such as joining a virtual “lab” and adding their samples to the group (6/12, 50%). Of the 12, 4 (33%) expressed confusion about the “lab” groups, including who is supposed to join them and for what purpose, while 3 (25%) expressed concerns about the legitimacy of *patient-led* “labs” because they might relate to actual future research on their samples, and they worried about the potential for unmoderated, off-topic, and illegitimate activity, with 1 participant stating “It should not feel like social media. To me it should feel more like science.”

The “explore” screen, showing high-level information about the entire biobank collection of samples, prompted the most confusion. Of the 14 co-designers who were able to review this screen, 12 (86%) were confused by ≥1 terms on this page (eg, “consent” and “protocols”), and 2 (14%) suggested the addition of a glossary to help users understand. Other co-designers (3/14, 21%) incorrectly assumed that the “explore” page contained information about an individual’s samples instead of the entire collection.

Participants also generally misunderstood the intended features of the “profile” page. Of the 11 co-designers who reviewed this page, 7 (64%) expressed confusion or dislike of the badges feature, with concerns that they were “useless,” “not important,” or excessively similar to features on social media sites, which they did not like. Finally, 18% (2/11) of the co-designers expressed concerns about the visibility of individual profile data, similarly worried about the parallels to existing social media, where all users can see individual profile pages.

### Phase 2

#### App Prototype Development

Taking participant feedback from phase 1 activities, we developed a functional app prototype with 4 sections corresponding to the screen designs. The “biowallet” screens had been well received, and minimal changes in content were made. We removed the diagnosis code from the “biowallet” page to avoid triggering negative memories and emotions in users. We maintained sample organization by type (tissue, blood, urine, etc) to more closely resemble the structure of biobank data, where the type of sample and medium of preservation are more valuable than the collection date. However, to address participants’ desire for a chronological arrangement, we listed samples from the most recently collected to oldest *within* the sample type category. The “biowallet” page also showed a record of the users’ informed consent to join the biobanking protocol. Users could click on individual samples to find information such as size, location, date collected, freezer number, and more. A generic picture of the corresponding sample type (eg, a picture of urine crystals under a microscope) accompanied each sample. Users could see whether each sample had been used in a research protocol or whether it was still available in the biobank.

The “connect” feature included patient-led “labs” organized by diagnosis, as in the original designs. We added a second class of “researcher-led labs” based on individual researchers who had used the patients’ samples. Due to the structure of deidentification of the initial biobank data, the names of the researchers and details about their work were blinded, with just a numerical code representing individuals (eg, principal investigator 4); summary statistics of their work (eg, 6 protocols using 75 samples); and similar blinded, summary information about specific research protocols using patient samples (eg, “Your samples were used in protocol #4, which used 25 samples”). Importantly, displaying this information did not impose any additional workload or data input responsibilities on researchers. The patient-led and researcher-led “labs” featured some cross-referencing. A patient-led group with a shared diagnosis was linked to researcher “labs” studying this diagnosis and to protocols using samples with this diagnosis. Using existing databases, we sorted individual users into the appropriate patient-led and researcher-led “labs,” adding a third section, “My Labs,” where users could view just the groups of which they were members. These 2 new groups of “labs” were developed to satisfy phase 1 participants’ enthusiasm for learning about research both in general and specifically about their samples, while maintaining maximum interoperability with existing biobank databases and protocols.

We updated terminology on the “explore” page in response to widespread confusion expressed by participants at the design workshop. Rather than relying on institutional terms such as “informed consent,” we framed participation in the biobanking protocol as being a “member.” For each sample category, we clarified how many patients were represented and how many samples there were in response to participants’ confusion about whether they were looking at 1 person’s samples on the “explore” page or the entire biobank’s samples. The “explore” page contained expandable information about the entire biobank collection, organized by sample type (eg, breast tissue, blood, and urine).

On the “profile” page, users could view the deidentified code that links their account on the app to the biobank database, populating their sample information. Rather than showing a hypothetical set of badges that users might be incentivized to collect, which were not well received, we showed just 1 indicator of whether users had successfully been connected to their sample collection. On the “profile” page, we also developed short questionnaires about potential future research topics of interest. This feature served as proof of concept for how the app might be used to engage participants in research design or be used as a data collection tool.

#### Design Workshop 2

The second participatory design workshop aimed to further validate the clarity and value of the information presented in the app. In addition, we received feedback on the user experience of navigating the live app.

All 9 groups of patients participating in this workshop (consisting of 25 patients total) liked learning the information presented to them on the app. Of the 9 groups, 3 (33%) specifically mentioned the “real-time” nature of the information, with one-third (n=1, 33%) stating that they liked being able to “follow along” with research. Of the 9 groups, 5 (56%) noted that they would prefer or require more information before using the app, such as background materials or guided onboarding, to better understand the information presented; 7 (78%) wished for more information (eg, details about research protocols); and 4 (44%) wished for more capabilities (eg, a search bar or the ability to make their biospecimen data more or less visible to others).

Of the 9 groups, 7 (78%) noted that the app was easy to use. However, 7 (78%) of the 9 groups also noted usability and navigation as something they disliked. Of the 9 groups, 4 (44%) noted that the onboarding process—downloading the app, creating an account, logging in, and verifying the user’s identity—did not meet their expectations or was not liked; 6 (67%) did not like a design feature of the app (eg, the choice of icon and the order in which the screens appeared); and 3 (33%) listed unmet accessibility expectations, including the desire for use on a larger screen or the preference for a web platform rather than a mobile app.

Echoing concerns from the first workshop, of the 9 groups, 3 (33%) were dissatisfied with the lack of explicit security assurances or data use terms, while 1 (11%) liked the fact that users’ identities were not featured on the app and were instead represented by a unique deidentified code.

#### Cognitive Walkthroughs

Cognitive walkthroughs with 13 patients also gauged the usability of the app. The walkthroughs illuminated an onerous and confusing onboarding process, where participants had to create an account and submit information to link their biobank data, after which they received a notification and request for more information once their biospecimen data were populated in the app. For users who were participants in the biobank, the interim waiting period for biospecimen data was as long as 1 week, and users often mistakenly thought they had done something incorrectly. Nearly all walkthrough participants expressed confusion and uncertainty about the onboarding and biospecimen data verification process, even when completed successfully.

Once they successfully logged in and were able to view biospecimen data, most of the participants successfully described their biospecimen data, including how many of their samples were available for research. Those participants who were members of the biobank, viewing their own biospecimen data, were curious as to why their samples had not been used and expressed a willingness to take action to ensure that their samples were used in research (eg, by offering to donate more samples or to pool samples with other participants). Overall, the success rates of completing this task indicated the usability of the app. Lower usability, namely the inability to identify successful completion of the task, arose in specific contexts applicable only to those participants who were attempting to view their own biobank data. Of the 13 participants, 3 (23%) had consented to participate in biobanking, but none of their clinical biospecimens made it to the biobank. Thus, their “biowallet” page showed no samples, although their “membership” was verified. This was a source of confusion, and participants incorrectly assumed that they had made a mistake in the onboarding process.

Participants were able to correctly identify a protocol that had used their samples or that had studied their disease. These participants successfully navigated from the “biowallet” screen to the “connect” screen and correctly understood the information presented about the protocol they identified. Some of the participants (7/13, 54%) expressed disappointment in the limited amount of information presented about the protocol or the associated researcher. Consistent with the initial survey findings, participants were most interested in learning the details and outcomes of the research conducted on their samples or their disease. In sum, the cognitive walkthroughs showed the usability of and interest in the app and revealed the need to improve the onboarding process and to better explain less intuitive scenarios, such as having no banked samples as a consented biobanking participant. The walkthroughs also confirmed participant interest in learning what happened to their biospecimens and about research on banked biospecimens.

## Discussion

### Key Findings

Throughout the design process, participants indicated a strong interest in and demand for learning about biobanking. They found intuitive value in the ability to track their own biospecimens. Indeed, participants positively received almost all of the information presented and commonly expressed a desire for more. In all phases, participants were most interested in learning about the research being conducted on their disease or specifically using their biospecimens, including the results of this research. They noted that they were not able to find this information on their own and were not able to satisfy general curiosity about research by participating in existing patient advocacy and support groups. These findings add to existing literature confirming public interest in greater transparency and engagement in biomedical research [32-36].

Mixed responses about the usability of the app demand further research and attention. Overall, participants in all stages were interested in using the app, although the content and presentation of information needed improvement. Some of the participants sought language and design similar to that of their medical records. Presenting research information in this way may make it more intuitively understandable but may also contribute to therapeutic misconception by misleading participants into believing that the research information and participation are of a similar clinically actionable quality. Thus, careful onboarding and framing of research information must ensure participant understanding and offer opportunities to overcome confusion.

Participants expressed a demand for a “legitimate” platform that did not suffer from what they saw as the risks and ills of social media: data misuse, a lack of privacy, and the potential for negativity or misinformation. While mobile phones and apps are widely used [37], concerns about mobile apps may make them an unacceptable platform for some. An additional facet of building trust with participants can be achieved through transparency surrounding how their biospecimens are being used. An interview participant expressed the following:

The biggest question that I have and other people have even when we’re asked you know, would you donate organs...is...the lack of knowledge about what it’s being used for.

This approach to rebuilding trust reflects ongoing movements in biomedical research aimed at improving transparency and accountability, such as interactive and accessible “data walks” designed to engage communities and stimulate dialogue [38].

However, the de-identification of biospecimens presents challenges for direct transparency and engagement. Other challenges exist as well—meaningful transparency and engagement with patients is time and labor intensive [39,40]. Even when community engagement is required or encouraged, there are few tools or guidelines for success, and interactions may be transient and transactional [40]. Biobanking and scientific research are deeply technical disciplines that historically have minimally interfaced with the public, and require extensive time, skill, and technology to translate into accessible, meaningful plain language [39]. Finding relevant patient communities and sustaining meaningful, collaborative relationships with them might prove beneficial to biobanks, or scientists themselves, but this is not required, supported, or incentivized by traditional academic reward systems or many institutions [41-43]. Engaging patients in biobanking might fulfill obligations of respect; however, evidence of its long-term impact on research participation, understanding, and attitudes is the subject of ongoing work [40].

Our experience confirms the need for specialized skills and dedicated time and resources to deliver transparency and engagement effectively and safely. New technologies, eg blockchain and generative artificial intelligence, are essential for these endeavors to be more efficient and scalable, but their development remains time and labor intensive. Participants were largely unfamiliar with biobanking, research processes or related jargon. Thoughtful, readily digestible and personalized education about each unique research context may be a critical aspect of the app onboarding process. Participants were confused about less intuitive but common scenarios, such as having consented to biobanking but having no samples in the bank. While this confusion may stem from a lack of recall or understanding of the initial informed consent for biobanking, delivering follow-up information about actual participation (or lack thereof) presents new risks of misunderstanding that must be managed.

While our study focused on patients with breast cancer, the demand for transparency and engagement may extend to other medical fields. We found that the survey participants (61/68, 90%) were most interested in learning the outcomes of research using their tissue samples. Thus, conditions with active research and strong patient interest in new treatment paradigms, such as additional cancer types and sickle cell disease, are potential areas where this platform may be effective. Further research is needed to assess the feasibility, acceptability, and effectiveness of this platform across diverse medical contexts and patient demographics.

Finally, in light of the strong reception and demand by participants, it must be acknowledged that returning deidentified, individual-level research information to participants is uncommon and unaccommodated by existing paradigms. Existing data architectures are built for a research enterprise from which participants are intentionally excluded. Working to disrupt the paradigm by allowing participants to view research data and activities presented both technological and regulatory implications. While participants were unaccustomed to receiving this type of information and, as a result, sometimes struggled to understand it, biobanks were also unaccustomed to sharing this type of information and, as a result, were sometimes hesitant to do so. Any technological tool aiming to enable transparency and engagement in research must be acceptable to all stakeholders. Careful and inclusive co-design is imperative.

### Limitations and Next Steps

The patient population we engaged in this work consisted of only women due to the nature of breast disease. Our participants were mostly White (64/94, 68%) and were more educated and had higher incomes than the national average, although they more closely resembled local demographics. Our participant population may also reflect self-selection bias—those interested in learning about research on their biospecimens or in general may have been more likely to participate in any research, including ours. Our technology was intended for piloting among this group and was therefore tailored to their preferences. For these reasons, further research will be necessary to validate interest in and usability of the technology in other patient populations (eg, men and healthy individuals).

Moreover, most of the patients we engaged with throughout all stages of design and development were unfamiliar with the term “biobank.” This underscores a key challenge associated with delivering an accessible and patient-friendly biobanking app. This variation in patient knowledge regarding biobanking, even among those who already consented to biospecimen donation, limits the ability to fully capture and address diverse patient preferences.

Across the surveys and workshops, questions and app content were iterated upon as app screens were finalized, new topics were raised by participants, and data saturation was reached through survey responses and follow-up interviews. As a result, the sample size of participants exposed to specific materials varied, leading to disproportionately lower sample sizes for certain areas of inquiry. Additional work is needed to investigate a larger sampling of patients’ design preferences. New technologies can facilitate meaningful transparency in biomedical research to patients in a personalized yet efficient way. This research showed that patients desire transparency and revealed the specific information they were interested in learning about their biospecimens. By deeply engaging patients and other stakeholders in participatory methods, siloed and specialized research data can be translated into meaningful patient engagement. We worked to design a mobile app that allows patients with breast cancer to track and learn about their donated biospecimens. Surveys, interviews, design workshops, and cognitive walkthroughs helped to make the app maximally valuable and usable for patients while maintaining efficient integration with existing data assets.

By creating this decentralized biobanking app, we built a platform upon which methods of transparency and engagement can be tested in subsequent pilots; for example, further research can measure the app’s impact on patients’ willingness to consent to biobank research, to remain enrolled and engaged longitudinally, and to actively participate in community-engaged research.

## References

[1] Boyer GJ, Whipple W, Cadigan RJ, Henderson GE (2012). Biobanks in the United States: how to identify an undefined and rapidly evolving population. Biopreserv Biobank.

[2] Henderson GE, Cadigan RJ, Edwards TP, Conlon I, Nelson AG, Evans JP, Davis AM, Zimmer C, Weiner BJ (2013). Characterizing biobank organizations in the U.S.: results from a national survey. Genome Med.

[3] Tarling TE, Byrne JA, Watson PH (2022). The availability of human biospecimens to support biomarker research. Biomark Insights.

[4] Li Y, Tang P, Cai S, Peng J, Hua G (2020). Organoid based personalized medicine: from bench to bedside. Cell Regen.

[5] Barnes RO, Watson PH (2020). Precision medicine: driving the evolution of biobanking quality. Healthc Manage Forum.

[6] Olson JE, Bielinski SJ, Ryu E, Winkler EM, Takahashi PY, Pathak J, Cerhan JR (2014). Biobanks and personalized medicine. Clin Genet.

[7] Zhou Z, Cong L, Cong X (2021). Patient-derived organoids in precision medicine: drug screening, organoid-on-a-chip and living organoid biobank. Front Oncol.

[8] Castillo-Pelayo T, Babinszky S, LeBlanc J, Watson PH (2015). The importance of biobanking in cancer research. Biopreserv Biobank.

[9] Warner TD, Weil CJ, Andry C, Degenholtz HB, Parker L, Carithers LJ, Feige M, Wendler D, Pentz RD (2018). Broad consent for research on biospecimens: the views of actual donors at four U.S. medical centers. J Empir Res Hum Res Ethics.

[10] Sobel ME, Dreyfus JC, Dillehay McKillip K, Kolarcik C, Muller WA, Scott MJ, Siegal GP, Wadosky K, O'Leary TJ (2020). Return of individual research results: a guide for biomedical researchers utilizing human biospecimens. Am J Pathol.

[11] Tomlinson T, De Vries RG (2019). Human biospecimens come from people. Ethics Hum Res.

[12] Rothstein MA (2010). Is deidentification sufficient to protect health privacy in research?. Am J Bioeth.

[13] Gross MS, Hood AJ, Rubin JC, Miller RC Jr (2022). Respect, justice and learning are limited when patients are deidentified data subjects. Learn Health Syst.

[14] Mamo N, Martin GM, Desira M, Ellul B, Ebejer JP (2020). Dwarna: a blockchain solution for dynamic consent in biobanking. Eur J Hum Genet.

[15] Gross MS, Miller RC (2019). Ethical implementation of the learning healthcare system with blockchain technology. Blockchain Healthc Today.

[16] Aguayo GA, Goetzinger C, Scibilia R, Fischer A, Seuring T, Tran VT, Ravaud P, Bereczky T, Huiart L, Fagherazzi G (2021). Methods to generate innovative research ideas and improve patient and public involvement in modern epidemiological research: review, patient viewpoint, and guidelines for implementation of a digital cohort study. J Med Internet Res.

[17] (2021). The James Lind Alliance guidebook. James Lind Alliance.

[18] Barony Sanchez RH, Bergeron-Drolet LA, Sasseville M, Gagnon MP (2022). Engaging patients and citizens in digital health technology development through the virtual space. Front Med Technol.

[19] Antoine-LaVigne D, Hayes T, Fortenberry M, Ohikhuai E, Addison C, Mozee S Jr, McGill D, Shanks ML, Roby C, Jenkins BW, Tchounwou PB (2023). Trust and biomedical research engagement of minority and under-represented communities in Mississippi, USA. Int J Environ Res Public Health.

[20] Scharff DP, Mathews KJ, Jackson P, Hoffsuemmer J, Martin E, Edwards D (2010). More than Tuskegee: understanding mistrust about research participation. J Health Care Poor Underserved.

[21] Kass NE, Faden RR (2018). Ethics and learning health care: the essential roles of engagement, transparency, and accountability. Learn Health Syst.

[22] Hood A, Macis M, Cervantes DM, Bear T, Khan J, Atkinson JM, Lee A, Gross M Privacy, policy, and profits: survey of patient values and preferences for research on de-identified biosamples. Advance.

[23] Gross M, Miller RC (2021). Protecting privacy and promoting learning: blockchain and privacy preserving technology should inform new ethical guidelines for health data. Health Technol.

[24] Gross MS, Hood AJ, Miller RC Jr (2021). Nonfungible tokens as a blockchain solution to ethical challenges for the secondary use of biospecimens: viewpoint. JMIR Bioinform Biotechnol.

[25] Spinuzzi C (2005). The methodology of participatory design. Tech Commun.

[26] Cavagnuolo M, Capozza V, Matrella A (2022). The walkthrough method: state of the art, innovative aspects, and application fields. Handbook of Research on Advanced Research Methodologies for a Digital Society.

[27] Whimsical homepage. Whimsical.

[28] Adalo homepage. Adalo.

[29] Eysenbach G (2004). Improving the quality of web surveys: the Checklist for Reporting Results of Internet E-Surveys (CHERRIES). J Med Internet Res.

[30] Flutter homepage. Flutter.

[31] Sanchez W, Linder L, Miller RC, Hood A, Gross MS (2024). Non-fungible tokens for organoids: decentralized biobanking to empower patients in biospecimen research. Blockchain Healthc Today.

[32] Jones RD, Krenz C, Griffith KA, Spence R, Bradbury AR, De Vries R, Hawley ST, Zon R, Bolte S, Sadeghi N, Schilsky RL, Jagsi R (2021). Governance of a learning health care system for oncology: patient recommendations. JCO Oncol Pract.

[33] Peppercorn J, Campbell E, Isakoff S, Horick NK, Rabin J, Quain K, Sequist LV, Bardia A, Collyar D, Hlubocky F, Mathews D (2020). Patient preferences for use of archived biospecimens from oncology trials when adequacy of informed consent is unclear. Oncologist.

[34] Sanderson SC, Brothers KB, Mercaldo ND, Clayton EW, Antommaria AH, Aufox SA, Brilliant MH, Campos D, Carrell DS, Connolly J, Conway P, Fullerton SM, Garrison NA, Horowitz CR, Jarvik GP, Kaufman D, Kitchner TE, Li R, Ludman EJ, McCarty CA, McCormick JB, McManus VD, Myers MF, Scrol A, Williams JL, Shrubsole MJ, Schildcrout JS, Smith ME, Holm IA (2017). Public attitudes toward consent and data sharing in biobank research: a large multi-site experimental survey in the US. Am J Hum Genet.

[35] Kaufman DJ, Murphy-Bollinger J, Scott J, Hudson KL (2009). Public opinion about the importance of privacy in biobank research. Am J Hum Genet.

[36] D'Abramo F, Schildmann J, Vollmann J (2015). Research participants' perceptions and views on consent for biobank research: a review of empirical data and ethical analysis. BMC Med Ethics.

[37] State of mobile 2024. Sensor Tower.

[38] Fattah L, Johnson J, Clark U, Hess L, Palermo AG, Figueroa Acosta DM, Swartz TH (2024). Building trust and transparency in biomedical sciences through data walks. Nat Med.

[39] Coors ME, Westfall N, Zittleman L, Taylor M, Westfall JM (2018). Translating biobank science into patient-centered language. Biopreserv Biobank.

[40] Luger TM, Hamilton AB, True G (2020). Measuring community-engaged research contexts, processes, and outcomes: a mapping review. Milbank Q.

[41] Stires H, Bado I, Brown T, Carlson M, Chan IS, Echeverria GV, Ewald AJ, Lim B, Lloyd C, Maues J, Oesterreich S, Riter RN, Shanahan K, Welm AL, Newby J (2022). Improving the odds together: a framework for breast cancer research scientists to include patient advocates in their research. NPJ Breast Cancer.

[42] Klingler C, von Jagwitz-Biegnitz M, Baber R, Becker KF, Dahl E, Eibner C, Fuchs J, Groenewold MK, Hartung ML, Hummel M, Jahns R, Kirsten R, Kopfnagel V, Maushagen R, Nussbeck SY, Schoneberg A, Winter T, Specht C (2022). Stakeholder engagement to ensure the sustainability of biobanks: a survey of potential users of biobank services. Eur J Hum Genet.

[43] Spector-Bagdady K, De Vries RG, Gornick MG, Shuman AG, Kardia S, Platt J (2018). Encouraging participation and transparency in biobank research. Health Aff (Millwood).

